# Infection with Foamy Virus in Wild Ruminants—Evidence for a New Virus Reservoir?

**DOI:** 10.3390/v12010058

**Published:** 2020-01-03

**Authors:** Magdalena Materniak-Kornas, Martin Löchelt, Jerzy Rola, Jacek Kuźmak

**Affiliations:** 1 Department of Biochemistry, National Veterinary Research Institute, 24-100 Pulawy, Poland; jkuzmak@piwet.pulawy.pl; 2 German Cancer Research Center DKFZ, Program Infection, Inflammation and Cancer, Division of Viral Transformation Mechanisms, 69120 Heidelberg, Germany; m.loechelt@dkfz.de; 3 Department of Virology, National Veterinary Research Institute, 24-100 Pulawy, Poland; jrola@piwet.pulawy.pl

**Keywords:** foamy viruses, BFV, wild ruminants, European bison, red deer, roe deer, fallow deer, seroreactivity, inter-species transmission

## Abstract

Foamy viruses (FVs) are widely distributed and infect many animal species including non-human primates, horses, cattle, and cats. Several reports also suggest that other species can be FV hosts. Since most of such studies involved livestock or companion animals, we aimed to test blood samples from wild ruminants for the presence of FV-specific antibodies and, subsequently, genetic material. Out of 269 serum samples tested by ELISA with the bovine foamy virus (BFV) Gag and Bet antigens, 23 sera showed increased reactivity to at least one of them. High reactive sera represented 30% of bison samples and 7.5% of deer specimens. Eleven of the ELISA-positives were also strongly positive in immunoblot analyses. The peripheral blood DNA of seroreactive animals was tested by semi-nested PCR. The specific 275 bp fragment of the *pol* gene was amplified only in one sample collected from a red deer and the analysis of its sequence showed the highest homology for European BFV isolates. Such results may suggest the existence of a new FV reservoir in bison as well as in deer populations. Whether the origin of such infections stems from a new FV or is the result of BFV inter-species transmission remains to be clarified.

## 1. Introduction

Foamy viruses (FVs), also known as spumaviruses, are the least known subfamily of *Retroviridae* [1]. Some features of their replication pathway and complex genomic organization distinguish them from other retroviruses [2,3]. Infections with FVs are persistent with sustained antibody response against viral antigens and the presence of viral DNA in leukocytes [4]. The most likely routes of FV transmission are via the transfer of blood and saliva and social interactions [3,5,6,7]. Over the last 60 years, FVs have been isolated and described in different species of non-human primates (Simian FVs (SFVs)) [8], as well as in cattle (Bovine FV (BFV), in the past also called bovine syncytial virus (BSV)) [9,10], cats (Feline FV (FFV)) and horses (Equine FV (EFV)) [3,11]. Several other non-primate FVs have been reported as having been isolated or simply described in sea lions, leopards, sheep, goats, hamsters, and American bison on the basis of cross-antigenicity with known FV, specific cytopathic effects or electron microscopy analyses [10,12,13,14,15,16]. Although FVs can be commonly isolated from infected animals, no disease has been associated with infections and, therefore, FVs are recognized as apathogenic on their own [17,18]. This lack of pathogenicity contrasts strongly with the cytopathic effects seen in vitro in infected cell cultures, with the appearance of “foamy-like” syncytia [17,19]. Based on the detection of diverse SFVs in simian-exposed humans, many studies have been focused on the inter-species transmission of FVs from simian and non-simian FVs [18,19]. While infections of humans by FVs from different simians and non-human primates are well evidenced, little is presently known about the possibility of such inter-species transmission caused by FVs of live-stock animals. Since BFV is highly prevalent within cattle populations [3,7,20], special attention should be paid to the possible involvement of BFV in inter-species transmission, especially regarding free-ranging wild ruminants. This is a very important and pertinent issue, owing to increasing human impact on the environment, globalization, and the establishment of breeding of some wild ruminants posing new threats including the uncontrolled transmission of infectious agents into wildlife [21,22]. There are many examples of highly prevalent life-stock viral pathogens crossing species barriers into wild ruminants, including bovine respiratory viruses like parainfluenza virus (BPIV-3), bovine adenovirus (BAdV), or bovine respiratory syncytial virus (BRSV) infecting European bison (*Bison bonasus*) in Poland [23]. The most important alphaherpesvirus, bovine herpesvirus 1 (BoHV) have also been reported to infect almost 40% of cervids in Poland [24], and a low percentage of the bison population [25]. Inter-species infections with ruminant retroviruses have been also reported previously: Bovine leukemia virus (BLV) infections have occasionally been described in European bison [25] or alpaca (*Vicugna pacos*) [26], while small ruminant lentiviruses (SRLV) infections have been found in Rocky Mountain goats (*Oreamnos americanus*) [27], Passirian goat in northen Italy [28] and recently in red deer (*Cervus elaphus*) and muflon (*Ovis aries musimon*) in Spain [29]. All reported cases are most likely due to the spill-over from domestic animals, acquired similarly to the well documented case of SRLV infection of endangered wild ibex (*Capra ibex*) in the French Alps, which was probably a result of sharing grazing grounds with a small herd of heavily infected goats [30].

The goal of the current study was the detection of antibodies and genetic material of BFV or a related FV in blood samples collected from free-ranging wild ruminants in Poland in order to address questions related to inter-species transmissions and altered pathogenicity in the new host or as part of a changed virome/microbiome.

## 2. Materials and Methods 

### 2.1. Animal Samples 

The samples used in this study came from 269 wild ruminants (suborder: *Ruminantia*, within the order of even-toed ungulates, *Artiodactyla*). Out of those, 256 samples were collected from cervids (family of *Cervidae*) including red deer (*Cervus elaphus*, n = 134), roe deer (*Capreolus capreolus*, n = 103), or fallow deer (*Dama dama*, n = 19), and 13 from free-ranging bovides (family of *Bovidae*), the highly endangered European bison (*Bison bonasus*). The serum samples of European bison and 18 fallow deer had been deposited as archival samples in the Departments of Biochemistry and Virology, NVRI, respectively. Whole blood samples were collected from the main vein or aorta of red deer and roe deer mainly as blood clots during seasonal hunting. All specimens were collected during the 2009/2010 hunting season. Blood clots were squeezed through sterile gauze and centrifuged for 15 min. at 3000 rpm. Obtained supernatants were collected for serological testing and pellets containing blood cells were washed twice with PBS and frozen at −70 °C until DNA preparation.

### 2.2. DNA Preparation

Total DNA was extracted from pelleted blood cells using the DNeasy Blood & Tissue Kit (Qiagen, Hilden, Germany) following the manufacturer’s instructions. DNA concentrations and the 260 nm/280 nm ratio were measured spectrophotometrically using GeneQuant (GE Healthcare, Warsaw, Poland) and stored at −20 °C until use. The DNA quality of selected samples was tested using capillary electrophoresis with highly sensitive gel (Fragment Analyser, Agilent).

### 2.3. Antibody Detection

GST (glutathione S-transferase) capture ELISAs were performed to examine the antibody response to BFV proteins in sera of wild ruminants using a well-established and validated generic GST-ELISA for domestic cattle as described by Romen and co-workers [7]. In short, 96-well microtiter plates (Thermo Labsystems, Dreieich, Germany) were coated with glutathione casein, blocked with 0.2% (*w*/*v*) casein and 0.05% (*v*/*v*) Tween 20 in PBS (blocking buffer), and then incubated with cleared *E. coli* lysates at a concentration of 0.25 μg/μL (total lysate in blocking buffer) containing the GST-tag or GST-X-tag fusion proteins (X = BFV-Gag, BFV-Bet, or BFV-Env). For pre-absorption of GST-binding antibodies, all sera were incubated at a dilution of 1:100 in a blocking buffer containing 2 μg/μL total lysate of a GST-tag expressing *E. coli* culture prior to application on the coated plates. After pre-absorption serum samples were incubated for 1 h at RT in the coated ELISA plate wells, washed, and incubated for 1 h at RT with Protein G—peroxidase conjugate (Sigma, 1:10,000 dilution). Protein G has a broad binding capacity for ruminant IgG [31]. TMB (Tetramethylbenzidine, Sigma, Poznan, Poland) was added as a substrate. For each serum, the absorbance of the GST-tag was determined and subtracted from the absorbance with the GST-X-tag protein to calculate the specific reactivity against the BFV antigens. Optical density (OD) measurements were done in duplicates and antibody levels were expressed as average net OD. As positive and negative internal controls, the pool of serum samples from five BFV naturally infected cows and five uninfected animals, diagnosed by GST-ELISA and PCR tests [32], were used at 1:100 dilutions.

Due to the lack of positive and negative controls from wild ruminants, cut-off values were calculated from the ELISA results for BFV Gag and Bet antigens obtained for cervids and European bison, excluding 3 outliers of bison origin and 9 outliers of cervid origin. These criteria resulted in 10 samples from European bison and 247 from cervids. The calculation was done in two ways: a less stringent cut-off was calculated as 1 × (mean + 3SD) and a highly stringent one as 2 × (mean + 3SD), which provided two cut-off values for each antigen, both methods are commonly used for diagnostic ELISA tests where defined negative and positive controls from the species tested are not available [33].

### 2.4. Western Blotting Analysis

Cf2Th cells (canine fetal thymus cells, Cat. No. 90110521, European Collection of Authenticated Cell Cultures (ECACC), UK) were co-cultured with BFV100-infected Cf2Th cells in a proportion of 10 to 1 and grown in DMEM, supplemented with 10% fetal bovine serum in the 5% CO_2_ atmosphere. Three days after infection, when the cytopathic effect appeared, cells were lysed using a CHAPS buffer (0.5 M EDTA, 1 M Tris HCL pH 8.8, 100 mM NaCl, 0.5 M CHAPS, 0.5 M sodium deoxycholate; Sigma, Poznan, Poland). Uninfected Cf2Th cells were grown under the same condition. Of the total cell lysates, 10 µg of infected and uninfected control cells were separated by SDS-PAGE and served as the antigen for western blotting analyses (WB) [4]. Wild ruminant sera were used at 1:100 dilutions (*v*/*v* in 3% bovine albumin, 0.01% Tween 20, PBS) and Protein G–peroxidase conjugate (Sigma, Poznan, Poland) at 1:10,000 dilution. As positive and negative controls, the pools of serum samples from five BFV naturally infected cows and five uninfected animals, diagnosed by GST-ELISA and PCR tests [32], were used at 1:100 dilutions. ECL Plus reagents (GE Healthcare, Warsaw, Poland) were used for the detection of specifically bound antibodies. 

### 2.5. FVs DNA Detection, Cloning, and Sequencing

Semi-nested polymerase chain reaction (PCR) was performed using genomic DNA from blood cells of 16 red and roe deer selected based on high reactivity in ELISA and the availability of high DNA quality, which was confirmed using a capillary system with highly sensitive gel (Fragment Analyser, Agilent). A set of external primers: BFVpolF1: TGGGAAAACCAGGTCGGACATC, BFVpolR: TACGACATCTGCTGTAAACAATGC, FFVpolF1: TGGGGAGAATCAGGTGGGTCATA and FFVpolR: TACAACATCTCCAGTAAACAACCC, EFVpolF1: TGGGAAAATCAAGTGGGACATA, EFVpolR: TACAACATCTGCAGTAAATAAGGC and internal primers: BFVpolF2: ATGGACGCTGGAGGATGGTGTTAGAC, FFVpolF2: ATGGTCGCTGGAGAATGGTACTGGAC and EFVpolF2: ATGGACGATGGAGAATGGTACTGGAT (Genomed, Warsaw, Poland) were designed to 100% match the corresponding part of the *pol* gene encoding for reverse transcriptase of all known non-primates FVs, i.e., BFV (GenBank accession no.: NC_001831.1 ), FFV (GenBank accession no.: NC_039242.1), and EFV (GenBank accession no.: AF201902.1). The first amplification included 2 U of DyNazyme DNA polymerase (Thermo Fisher Scientific), 1× PCR buffer with 1.5 mM MgCl2, 0.2 µM of each primer, 0.4 mM of dNTP-mix (Thermo Fisher Scientific) and 1µg of genomic DNA. The temperature profile was as follows: initial denaturation at 94 °C for 3 min, denaturation at 94 °C for 45 s, annealing at 54 °C for 45 s, elongation at 72 °C for 1 min, and final elongation at 72 °C for 5 min. Semi-nested amplification was completed in similar conditions using 1/10 volume of the first PCR as a template. The expected size of the amplicon was 275 bp.

Another semi-nested PCR was performed using DNA from the blood of one selected red deer. The following primers (Genomed, Warsaw, Poland) were used to amplify the sequence located in the BFV *gag* gene (1139-1381 nt): Gag-1: GACGCAACAAACCAACCAC; Gag-2: GTTCTTGTCCGTATCGTTGTG [34] and BFVpolR: TACGACATCTGCTGTAAACAATGC. The first amplification was performed in the same conditions as described for the BFV *pol* reaction, but included Gag-1 and BFVpolR primers, while the semi-nested PCR was performed as described previously with Gag-1 and Gag-2 primers [4], but using 1/10 volume of first PCR as a template. The expected size of the semi-nested PCR product was 243 bp.

Nested PCR for BFV LTR-derived sequences was performed using genomic DNA from blood cells of selected deer (see earlier in this section). The following primers (MWG Biotech) within the BFV LTR region, mostly covering the U3 region and the beginning of the R region, were used in this study: BFV-LTR-1_S: TTACTTGCCCGGAGGATTGG, BFV-LTR-1_AS: TAGTGATCTGGAAGGTAAGC, BFV-LTR-2_S: CTTATGGATGGAGCCTTATGG, BFV-LTR-2_AS: CTTACCACAGCCTGGAAGTC. All primers were designed to 100% match all known BFV sequences (GenBank accession no.: U94514, GI 9629644, GI 22947830). The first reaction of the amplification included 2.5 U of *Taq* DNA polymerase (ThermoFisher Scientific), 1× PCR buffer with 1.5 mM MgCl2, 0.2 µM of each primer, 0.1 mM of dNTP-mix (ThermoFisher Scientific), and 1µg of genomic DNA. The first PCRs were performed with the following temperature profile: initial denaturation at 94 °C for 3 min, denaturation at 94 °C for 45 s, annealing at 52 °C for 45 s, elongation at 72 °C for 2 min, and final elongation at 72 °C for 10 min. Nested amplification was done at similar conditions, with the exception of the annealing step where the temperature was 54 °C for 45 s and using 1/10 volume of the first PCR as a template. The expected size of the amplicon was 874 bp.

The resulting amplicons were analyzed on 1% agarose gels, cloned into the pCR2.1-TOPO vector (Invitrogen), and sequenced from both sides according to the method of Sanger [35] by Genomed, Warsaw, Poland for the *pol* sequence and GATC (Konstanz, Germany) for the LTR sequences.

For bioinformatics analyses, primer sequences were removed from the cloned amplicons, which were aligned to the reference sequences of non-primate FVs available in GenBank (EFV—LC_381046.1, AF201902.1, NC_002201.1, BFV-US—NC_001831.1, BFV-3026—AY134750.1, BFV-100—JX307861.1, and BFV Riems—JX307862.1, as well as all available isolates of FFV—accession numbers are listed on the phylogenetic tree in Figure 5) using the Geneious alignment module within the Geneious Pro 5.3 software (Biomatters Ltd., Auckland, New Zealand). The alignment was submitted to the MEGA 6.0 version for the best model selection measured by the the Bayesian information criterion (BIC) and the corrected Akaike information criterion (AICc). According to the results Tamura 3-parameter with Gamma distribution [36] substitution model was applied in MEGA 6.0 to infer a phylogenetic tree using maximun likelihood method. The statistical confidence limits of the phylogram topologies were assessed with 1000 bootstrap replicates. The sequences obtained in this study were deposited in the GenBank database with the following accession numbers: MN630606-MN630611 for *pol* and MN630602–MN630605 for LTR sequences.

### 2.6. Statistical Analysis

Scatter plot analyses were performed to calculate the linear correlations of the net OD values obtained for Gag and Bet antigens in ELISA tests. Calculations and the generation of graphs were done using STATISTICA ver. 10 (StatSoft, part of Dell Software, USA).

## 3. Results

### 3.1. Serological Screening of Wild Ruminants Samples

**Figure 1:**
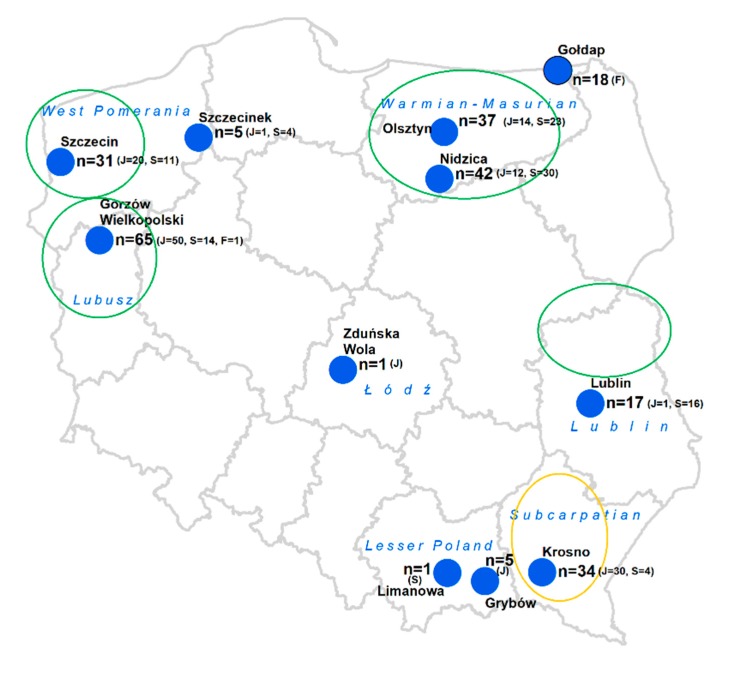
Geographic distribution of samples collected from wild deer in Poland. Blue dots show the areas of the samples collection which correspond to closest cities, n—the number of samples collected in the particular areas. F—no. of fallow deer, J—no. of red deer, S—no. of roe deer; green rings—the major regions of dairy cow production in big farms; orange ring—regions with moderate dairy cow production, mainly in small family farms.

The study included 269 serum samples from wild ruminants collected in different parts of Poland (Figure 1). The samples originated from cervids such as red deer (*Cervus elaphus*, n = 134), roe deer (*Capreolus capreolus*, n = 103), and fallow deer (*Dama dama*, n = 19), as well as free-ranging bovides, the highly endangered European bison (*Bison bonasus*, n = 13). Serum samples were assayed for the reactivity toward BFV Gag, Bet, and Env-SU antigens using GST-capture ELISA as previously described [7]. The observed net OD values for the Gag antigen ranged between 0.001–1.246 and between 0.001–1.339 for Bet in cervid samples. The overall reactivity of bison samples was lower and ranged between 0.056–0.660 for Gag and 0.087–0.590 for Bet. The reactivity to the Env-SU antigen was very low, comparable with the background, and was therefore not further considered as the GST-tagged Env has also been shown in previous studies to be of low diagnostic value [7].

**Figure 2:**
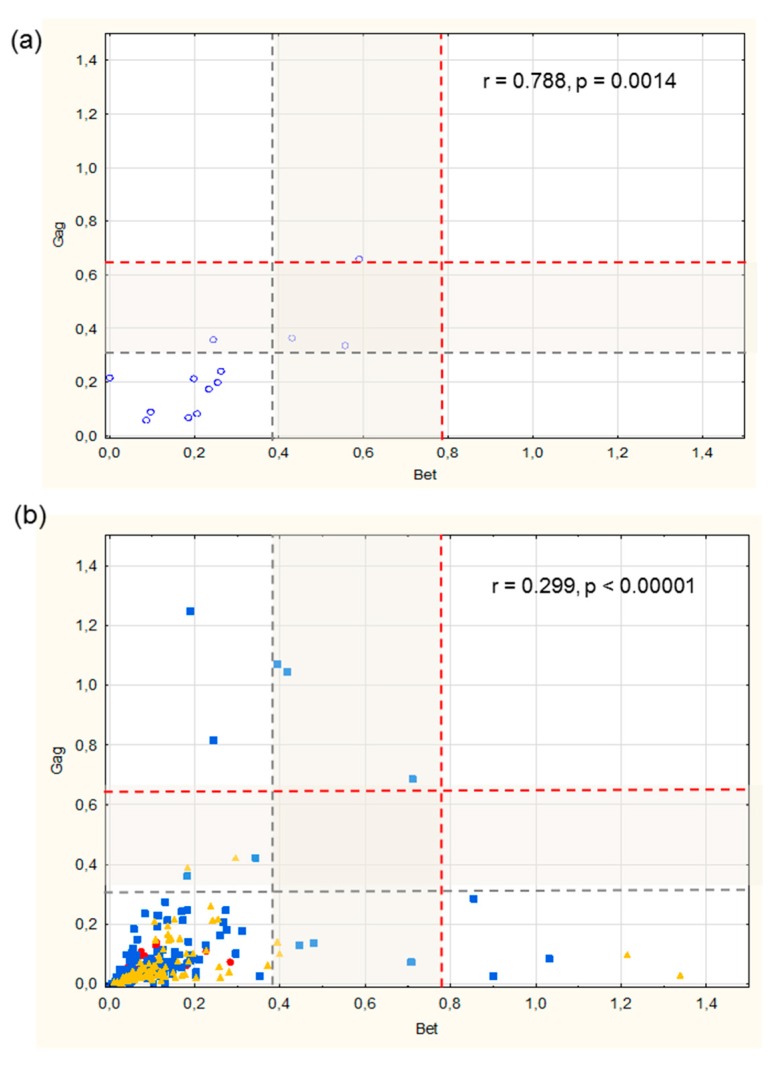
Distribution of Gag and Bet serological reactivity presented as scatter plots: (**a**) serum samples of bison origin, (**b**) serum samples of cervid origin. Each point represents a data pair of an individual serum of the following origins: blue dot—European bison, red dot—fallow deer, blue square—red deer, and yellow triangle—roe deer. Dashed lines, grey and red, indicate the cut-off values calculated in two ways grey—cut-off = 1 × (mean + 3SD), red—cut-off = 2 × (mean + 3SD). The grey boxes between the dashed lines indicate the grey zones of inconclusive ELISA results. The upper right sector shows double-positive, the lower-left double-negative, the lower-right sector displays sera positive for Bet only, and the upper left sector represents sera positive for Gag exclusively. The correlation coefficient (r) and *p*-value (p) are indicated in the graphs.

Due to the differences of overall reactivity, scatter plot analyses of the net OD values for Gag and Bet antigens were separately performed for European bison (Figure 2a) and the different deer species (Figure 2b), which showed that the correlation between the seroreactivity to both antigens was strong in the population of bison, while in deer, it was weaker.

Cut-off values were calculated for Gag and Bet separately, using two methods commonly used in serodiagnostics for the different tests and animal populations (see Materials and Methods, grey and red dashed lines) (Figure 2). This approach distinguished between very high reactive sera, as well as those assessed as inconclusive with reactivity in the grey intermediate zone between the two independently calculated cut-off values (Figure 2). 

This analysis indicated that seven samples showed clearly high reactivity in comparison to other samples only in deer populations, while the reactivity of another 16 samples was in the intermediate grey zones. Out of these 23 samples with high or intermediate reactivity, four were from European bison, representing 30% of bison samples tested. The remaining 19 samples represent 7.4% of deer specimens tested, including six from roe deer and 13 from red deer origin. Interestingly, all highly reactive deer samples came from six locations, with seven samples (7/256, 2.7%) from Gorzów Wielkopolski in the western part of Poland, and six samples (6/256, 2.3%) from Nidzica in the north of the country (Figure 1, Table 1). Three samples from bison and three from red deer showed high reactivity to both, Gag and Bet antigens. One bison and six deer samples reacted strongly with Gag only (4 from red deer and 2 from roe deer), while ten samples were reactive to Bet antigen (6 from red deer and 4 from roe deer). 

**Figure 3:**
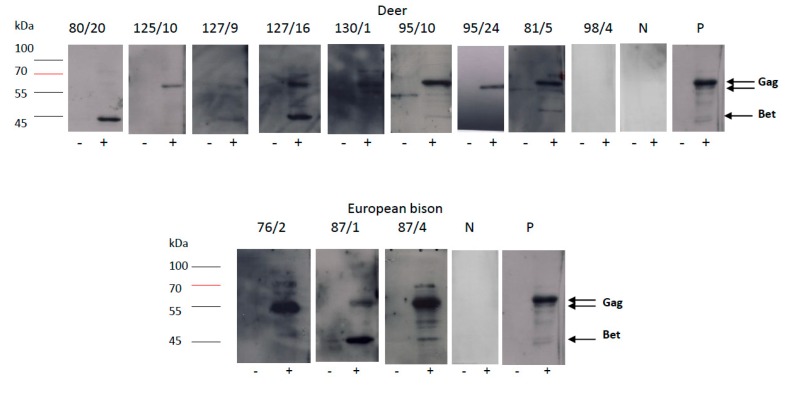
Detection of foamy virus (FV)-specific antibodies by immunoblotting analysis with a cellular antigen in representative serum samples of deer and European bison; (−) lane with uninfected Cf2Th cells lysate as antigen, (+) lane with Cf2Th/BFV100 cells lysate as antigen; P—BFV positive control serum, N—BFV negative serum.

To verify the ELISA results, 15 samples with reactivity to at least one BFV antigen were subsequently tested by western blotting assay (WB) with lysates of BFV_100_-infected Cf2Th cells, used as an antigen. Uninfected Cf2Th cells were used as a control antigen. Specific reactivity against Gag results in the presence of a double band at about 60/58 kDa and, for Bet, a single band at approximately 46 kDa is characteristic (own unpublished study). Out of the 15 samples tested, eleven samples showed strong reactivity to the BFV cellular antigen (Figure 3) including three ELISA-positive samples from bison, while three samples from deer showed only very faint bands in WB, marked by +/-, in Table 1. The pattern of bison sera WB reactivity was very similar to the positive control, which contained pooled sera of naturally BFV-infected cows. In contrast, the pattern of deer reactivity was slightly different, especially for Bet reactivity, which was, in most cases, clearly weaker and even completely missing in animals 125/9 and 125/10. One sample, no. 98/4 showed no reactivity in WB.

### 3.2. Detection of FV DNA

**Figure 4:**
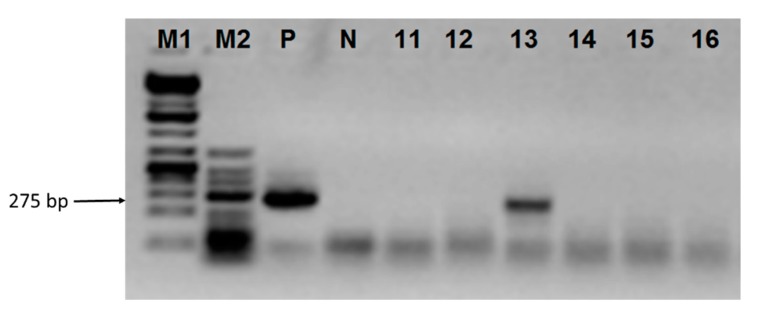
Electrophoretic analysis of semi-nested PCR amplification products. M1, Gene Ruler 1 kb Plus DNA Ladder; M2, GeneRuler Low Range DNA Ladder, Fermentas, **P**, positive control (blood DNA of calf experimentally inoculated with BFV), N, negative reaction control, samples no. 113/11–113-16.

**Figure 5:**
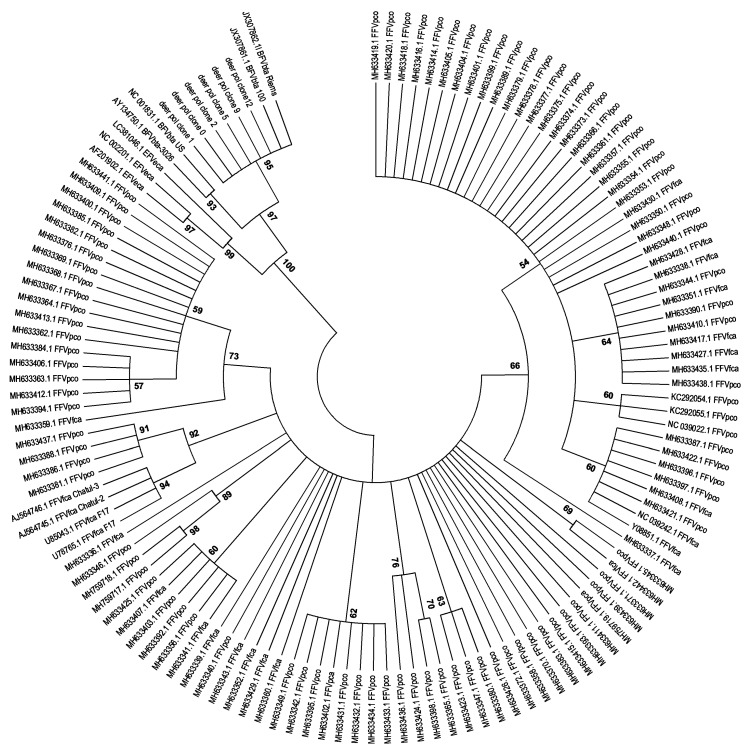
Phylogenetic tree inferred from the 275 bp amplicons of the *pol* region from deer 113/13 and extracted from the sequences of all BFV, EFV, and FFV isolates (the majority of the sequences) available in GenBank. The origin of FV isolates has been indicated as follows: BFVbta, cow (*Bos taurus*); EFVeca, horse (*Equus caballus*); FFVfca, cat (*Felis catus*); FFVpco, mountain lion (*Puma concolor*). All analyzed sequences were devoid of primer sequences. The tree was generated with MEGA 6.0 software by maximum likelihood method and presented with condensed branches.

We then aimed to detect FV-specific DNA in the total DNA extracted from the blood cells of ruminants with high reactivity towards BFV antigens, as determined by serology. A set of specific PCR primers was designed to match a conserved region of the *pol* gene encoding the BFV, EFV, and FFV reverse transcriptase. DNA extracted from blood cells of animals with serum reactivity towards BFV antigens was used as the template in semi-nested PCRs. Specific amplification was obtained for only one sample collected from red deer no. 113/13 (Figure 4). The 275 bp PCR product was cloned and sequenced. Alignment of sequences of six clones with all sequences of BFV, EFV, and FFV isolates available in GenBank showed the highest homology with the Polish BFV_100_ isolate and German BFV Riems, both representing the European clade of BFV [37] (97% identity for clone 0 and 100% for the other four clones) (Figure 5). Such a high similarity is comparable to the homology of FFV sequences of domestic cat and mountain lion origin.

**Figure 6:**
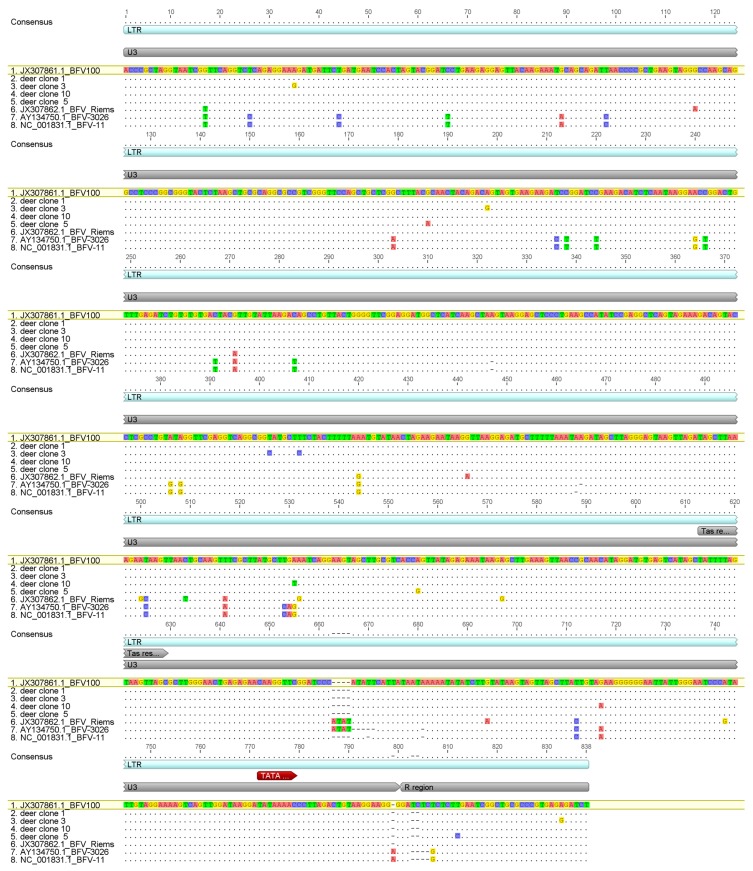
Alignment of the LTR clones sequences derived from red deer no. 113/13 with respective sequences extracted from BFV isolates available in GenBank.

Furthermore, a set of pan-BFV specific primers was used to amplify part of the LTR sequences from red deer no. 113/13. The LTR PCR amplicons were cloned, sequenced, and aligned to the respective sequences of BFV isolates available in GenBank (Figure 6). Unfortunately, no successful amplification was achieved when primers specific for BFV *gag* were used.

**Figure 7:**
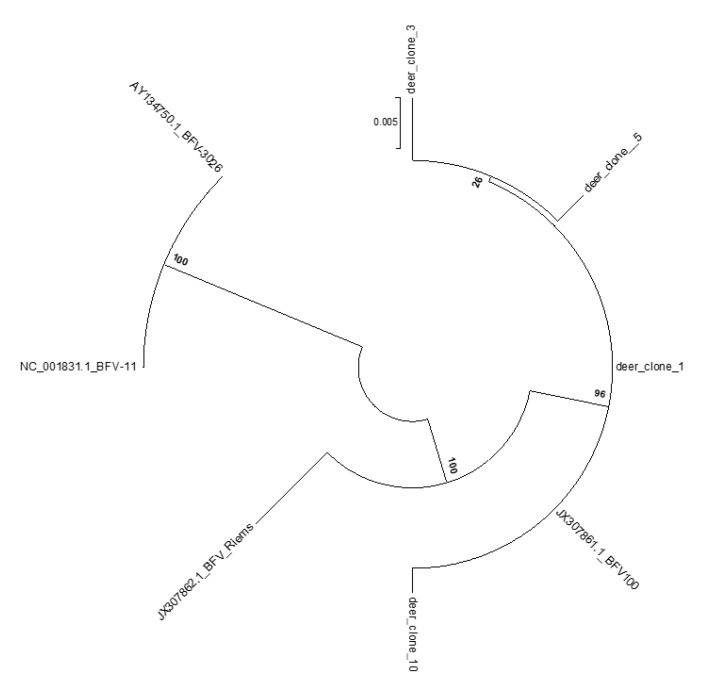
Phylogenetic tree inferred from the 874 bp sequence of the LTR region amplified from deer no. 113/13 and extracted from sequences of all BFV isolates available in GenBank. All analyzed sequences were devoid of primer sequences. The tree was generated with MEGA 6.0 software by maximum likelihood method. Bar—nucleotide substitutions per site.

Both phylogenetic analyses (Figure 5 and Figure 7) clearly show that the new sequences from European red deer are most closely related to the European clade of known BFV isolates [37]. In addition, LTR clones showed the highest similarity to the Polish BFV_100_ isolate (99.2% to 99.8% identity for analyzed clones) (Figure 6). The overall similarity for both sequences was very high and most changes were single transitions or transversion. Interestingly a deletion of two nucleotides at the LTR position 803/804 nt, the beginning of the R region of the LTR, was present in all clones from red deer, which is not present in any of the European clade BFV isolates.

## 4. Discussion

Most known FVs are highly prevalent in their hosts. For instance, the prevalence of BFV among cattle ranges between 7% and 50% of cattle worldwide, while in Poland it reaches over 30% (see for summary [10]). Transmission of BFV is suggested to occur through saliva, and, therefore, there is likely to be a risk of its spread through shared grazing areas or direct social or environmental contacts [5,6]. Serological investigation of sera from wild ruminants, presented in this study using BFV Gag and Bet antigens, clearly showed that about 8.5% of the sera reacted with at least one BFV antigen in a generic ELISA system. None of the tested samples reacted with the BFV Env antigen, which has already been shown to be an antigen with low diagnostic value in BFV-infected cattle [7,38]. Interestingly, about one fourth (3/13) of European bison sera analyzed specifically reacted with both BFV Gag and Bet in two independent serological assays. In contrast, only 9.7% of red deer and 5.8% of roe deer serum samples tested with the Gag and Bet ELISA and the WB based on the BFV-infected cells showed reactivity and it was often either against Bet or Gag but rarely (1.1%) against both antigens together. None of the fallow deer serum samples reacted with Gag or Bet antigens, which may be because of the low number of samples tested in comparison to red or roe deer. Highly BFV-related, conserved non-primate FV *pol* and LTR sequences were detected by diagnostic PCR in blood cell samples from one red deer, corresponding analyses could not be performed for bison due to the lack of DNA samples. 

If the strongly BFV-related sequences isolated from the BFV-reactive red deer 113/13 are also representative for the other deer scored seropositive, this would indicate that these animals have probably been infected with BFV derived from domestic cattle. Since contact between deer and grazing cattle is possible under the farming conditions in Poland, we assume that inter-species transmission from cattle to red and roe deer and possibly other deer species occurs at a low but consistent level similar to the transmission of SFVs to humans highly exposed to non-human primates [18,39,40,41]. In simian-to-human transmissions, aggressive behavior, biting, and blood exchange are the major route of infection, this is different in ruminants. In BFV, free and mixed grazing of cattle and deer in rural areas may lead to BFV transmission as has been well documented for small ruminant lentiviruses in France by Erhouma and others [30], where close contact between wild ibex and goats at pastures led to small ruminant lentiviruses infections in wild animals, or for BoHV-1 which was detected in cervid populations in Germany and in Poland [24,42]. BFV transmission through saliva has been described as the most frequent route of transmission in natural conditions [3,10]; however, our own studies showed that the isolation of BFV from saliva is possible only from a small percentage of animals [32]. Unpublished data also showed that, in contrast to SFV or FFV, BFV viral load in the saliva of naturally and experimentally infected cows is very variable, from quite high to undetectable in some BFV-positive animals, but the explanation for this phenomenon is still unclear. However, a similar scenario in the wild may limit the possibility of BFV transmission to wildlife animals. 

The low correlation of Bet and Gag reactivity in both deer species can also be taken as an indication for individual inter-species transmission events. These interspecies transmissions may result in attenuated or abortive replication, which could lead to incomplete or variable immune recognition of BFV antigens and thus a deviant pattern of immune responses compared to BFV in productively infected cattle [43]. In addition, the abrogation of the immune response after the transgression of the species barrier and adaptation to the new host has been reported for some viruses [43,44] and may explain the immune response pattern seen here in deer. Alternatively, wild-ranging red, roe, and fallow deer may be also exposed to currently unknown BFV-like viruses from other ruminants leading to unexpected and variant patterns of immune reactivity. This point may also explain the high number of PCR negative deer samples in contrast to the results of serological tests. The genetic differences of BFV-like viruses from different species could affect primer binding, leading to a lack of amplification, but, on the other hand, a very low number of BFV copies (below the sensitivity of PCR reaction reaching 10 copies) in peripheral blood of infected animals or the presence of any PCR inhibitors could also lead to negative PCR results. However, such discordance between the presence of BFV-specific antibodies and viral DNA may also result from the natural clearance of the productive BFV infection in heterologous hosts as was observed by Morin and co-workers [44] in experimental infection of calves with caprine arthritis-encephalitis virus.

The high prevalence (3 out of 13 animals tested) and overall tight correlation of reactivity against BFV Gag and Bet in European bison (see ELISA and WB data, Figure 2 and Figure 3) may indicate that a currently unknown BFV-like virus is endemic in bison. This may be similar to the isolation of a retrovirus similar to the bovine foamy virus from American bison, reported years ago by Amborski and co-workers [12]. While such a bison-specific FV is serologically related but distinct (comparably low signal intensities in the Gag and Bet ELISAs, [7,38]), it may also be genetically distinct from the BFV of cattle. In contrast to deer, the habitats of bison and domestic cattle do not overlap frequently since bison are kept in nature reserves, clearly reducing the chance of exposure to BFV from cattle. 

European bison are an endangered large European animal that was close to extinction at the beginning of the 20th century. Tight protection programs, especially in Poland and other countries [45], have led to the recovery of this iconic species at the price of severe inbreeding. Loss of genetic diversity within a species increases the susceptibility to infection with many pathogens, including bacteria, viruses, and parasites [23,46,47]. Under such conditions, even innocuous infectious agents like BFV may gain pathogenic potential, either on their own or as part of the microbiome/virome. This indicates a strong rationale for further investigations into old and emerging ruminant FVs. In addition, since only low numbers of bison have been studied here, further research that includes higher numbers of samples is warranted. These new studies should also go beyond serology and PCR-mediated DNA detection and should include the analysis of oral samples and milk for virus detection and isolation and maybe even encompass novel DNA detection methods based on deep sequencing.

In summary, we demonstrate clear evidence that free-ranging ruminants are exposed to and infected by FVs closely related to BFV. However, more studies are required to know whether the highly endangered, inbred, and iconic bison and the different deer species are infected by species-specific, novel FVs or by BFV of cattle origin crossing species barriers. 

## Figures and Tables

**Table 1:** Summary of Gag and Bet ELISA, western blotting (WB), and PCR results.

Sample No.	Species	OD Gag	OD Bet	OD Env	WB Gag	WB Bet	PCR	Area of Samples Collection
95/24	red deer	0.083	1.033	0.002	+	+/-	-	Gorzów Wlkp
113/11	red deer	0.13	0.447	0.002	n.t.	n.t.	n.t.	Gorzów Wlkp
113/13	red deer	0.074	0.709	0.008	+/-	+	+	Gorzów Wlkp
113/14	roe deer	0.102	0.399	0.003	n.t.	n.t.	n.t.	Gorzów Wlkp
125/9	red deer	1.07	0.395	0.018	+/-	-	-	Gorzów Wlkp
125/10	red deer	1.047	0.478	0.023	+	-	-	Gorzów Wlkp
95/10	red deer	0.286	0.855	0.001	+	+	-	Gorzów Wlkp.
81/05	red deer	1.246	0.192	n.t.	+	+	-	Krosno
98/04	roe deer	0.391	0.183	0	-	-	-	Lublin
80/05	roe deer	0.141	0.394	n.t.	n.t.	n.t.	-	Nidzica
80/16	roe deer	0.423	0.297	n.t.	n.t.	n.t.	n.t.	Nidzica
80/17	red deer	0.424	0.342	n.t.	n.t.	n.t.	n.t.	Nidzica
80/20	red deer	0.685	0.710	n.t.	+	+	-	Nidzica
80/24	roe deer	0.031	1.339	n.t.	+/-	+/-	-	Nidzica
80/32	red deer	0.36	0.182	n.t.	n.t.	n.t.	n.t.	Nidzica
91/15	red deer	0.138	0.48	n.t.	n.t.	n.t.	-	Olsztyn
130/1	roe deer	0.097	1.214	0.013	+	+/-	-	Olsztyn
127/9	red deer	0.026	0.901	0.028	+	+	-	Szczecin
127/16	red deer	0.915	0.246	0.093	+	+	-	Szczecin
76/01	Bison	0.359	0.245	nt	n.t.	n.t.	n.a.	n.a.
76/02	Bison	0.366	0.432	n.t.	+	-	n.a.	n.a.
87/01	Bison	0.338	0.556	n.t.	+	-	n.a.	n.a.
87/04	Bison	0.660	0.590	n.t.	+	+	n.a.	n.a.

n.t.—not tested, n.a.—not available. Areas of sample collection are indicated according to Figure 1.
